# Solid-phase electrochemical reduction of graphene oxide films in alkaline solution

**DOI:** 10.1186/1556-276X-8-397

**Published:** 2013-09-24

**Authors:** Wan J Basirun, Mehran Sookhakian, Saeid Baradaran, Mohammad R Mahmoudian, Mehdi Ebadi

**Affiliations:** 1 Department of Chemistry, University Malaya, Kuala Lumpur 50603, Malaysia; 2 Nanotechnology & Catalysis Research Centre, Institute of Postgraduate Studies, University Malaya, Kuala Lumpur 50603, Malaysia; 3 Department of Mechanical Engineering, Faculty of Engineering, University Malaya, Kuala Lumpur 50603, Malaysia; 4 Department of Chemistry, University of Farhangian, Tehran 15916, Iran

**Keywords:** Carbon materials, Thin films, FTIR, Raman

## Abstract

Graphene oxide (GO) film was evaporated onto graphite and used as an electrode to produce electrochemically reduced graphene oxide (ERGO) films by electrochemical reduction in 6 M KOH solution through voltammetric cycling. Fourier transformed infrared and Raman spectroscopy confirmed the presence of ERGO. Electrochemical impedance spectroscopy characterization of ERGO and GO films in ferrocyanide/ferricyanide redox couple with 0.1 M KCl supporting electrolyte gave results that are in accordance with previous reports. Based on the EIS results, ERGO shows higher capacitance and lower charge transfer resistance compared to GO.

## Background

Graphene, a one-dimensional carbon sp^2^-bonded compound is finding considerable attention in the development of advance nanomaterials. Chemically modified graphene is studied for their importance in biomedical sensors, composites, field-effect transistors, energy conversion, and storage applications due to its excellent electrical, thermal, and mechanical properties. Reduced graphene oxide (RGO) can be produced by the reduction of graphene oxide (GO) by various methods. High temperature annealing of GO above 1,000°C is an effective method to produce RGO [1]. Sodium borohydride [2] and hydrazine [3-5] are also acceptable chemical methods for the reduction of GO to produce the RGO. Among the methods to synthesize RGO are by chemical exfoliation of GO in propylene carbonate followed by thermal reduction [4,5]. Another method of reduction of GO is by using hydrohalic acids [6]. Nutrients such as vitamin C [7,8] and metallic element such as aluminum powder [9] are also viable reducing agents for the production of RGO from GO. Hydrothermal reduction is also an effective method for the reduction of GO to RGO [10]. Electrochemical reduction to produce RGO or better known as electrochemically reduced graphene oxide (ERGO) is considered a green method which offers safer procedures compared to other chemical methods of reduction without the use of dangerous chemicals such as hydrazine. A suspension of GO was evaporated on glassy carbon and used as an electrode and reduced by voltammetric cycling in 0.1 M Na_2_SO_4_ solution to produce ERGO films [11]. Electrochemical reduction of GO suspensions were also done in acidic media using phosphate buffer solution at pH 4 [12] and basic pH at 7.2 [13]. Direct electrochemical reduction of GO onto glassy carbon has also been reported [14] in sulfuric acid [15] and in NaCl solution [16]. Electrophoretic deposition of GO to produce ERGO is also an effective method to produced solid films of ERGO [17]. Several authors [11,13,14,18] have performed voltammetric cycling of exfoliated GO sheets from colloidal suspensions and found that electrochemical reduction for different functional groups in GO are dependent on the reduction potential. In this work, voltammetric cycling was used to electrochemically reduce GO films to ERGO in KOH solution.

## Methods

### Chemicals

All chemicals such as KOH, KCl, K_4_[Fe(CN)_6_], and K_3_[Fe(CN)_6_] were of Analar grade and procured from Sigma Aldrich (St. Louis, MO, USA).

### Synthesis of GO

GO was synthesized using a modified Hummers' method [19]. GO was dispersed in a beaker filled with distilled water and sonicated for 5 h. GO dispersion with a concentration of 0.3 mg cm^-3^ was poured on a graphite sheet in the jar and evaporated overnight in an oven at 60°C.

### Material characterization

Field emission scanning electron microscopy (FESEM) using a Quanta 200F instrument (FEI, Hillsboro, OR, USA), was used to capture the images of the evaporated GO and ERGO layers on the graphite sheet. Fourier transformed infrared (FTIR) spectroscopy was carried out using Spectrum 400 instrument while Raman spectroscopy was done with a Renishaw inVia Raman microscope (Wotton-under-Edge, UK) using (*λ* = 514 nm) laser excitation.

### Electrochemical methods

Cyclic voltammetry (CV) and electrochemical impedance spectroscopy (EIS) were done using a potentiostat / galvanostat, Autolab PGSTAT-302N from Ecochemie (Utrecht, the Netherlands). A general purpose electrochemical software installed in the computer interfaced with a USB card (USB_IF030) was used to run the CV experiments while frequency response analysis (FRA) software was used to run the EIS experiments. The CV and EIS experiments were done in a single compartment cell. A mercury oxide (Hg/HgO) reference electrode (RE) and graphite rod counter electrode (CE) was used in the voltammetric cycling for the reduction of GO films in 6 M KOH solution at a scan rate of 50 mV·s^-1^. The CV experiments performed in 6 M KOH solution and [Fe^II^(CN)_6_]^3-/4-^ redox couple in 0.1 M KCl supporting electrolyte were done on stationary electrodes. A two-electrode configuration was used in the EIS experiments using the working electrodes (WE), and a saturated calomel electrode (SCE) as the reference and counter electrode (RE-CE). The EIS measurements were performed over a frequency range of 100 kHz to 10 mHz, with an acquisition of 10 points per decade, and with a signal amplitude of 5 mV around the open circuit potential. Analysis of the impedance spectra was done by fitting the experimental results to equivalent circuits using the nonlinear least-square fitting procedure with the chi-squared value minimized to 10^-4^. All experiments were performed at room temperature 300 K.

## Results and discussions

### Voltammetric reduction of GO to ERGO

**Figure 1:**
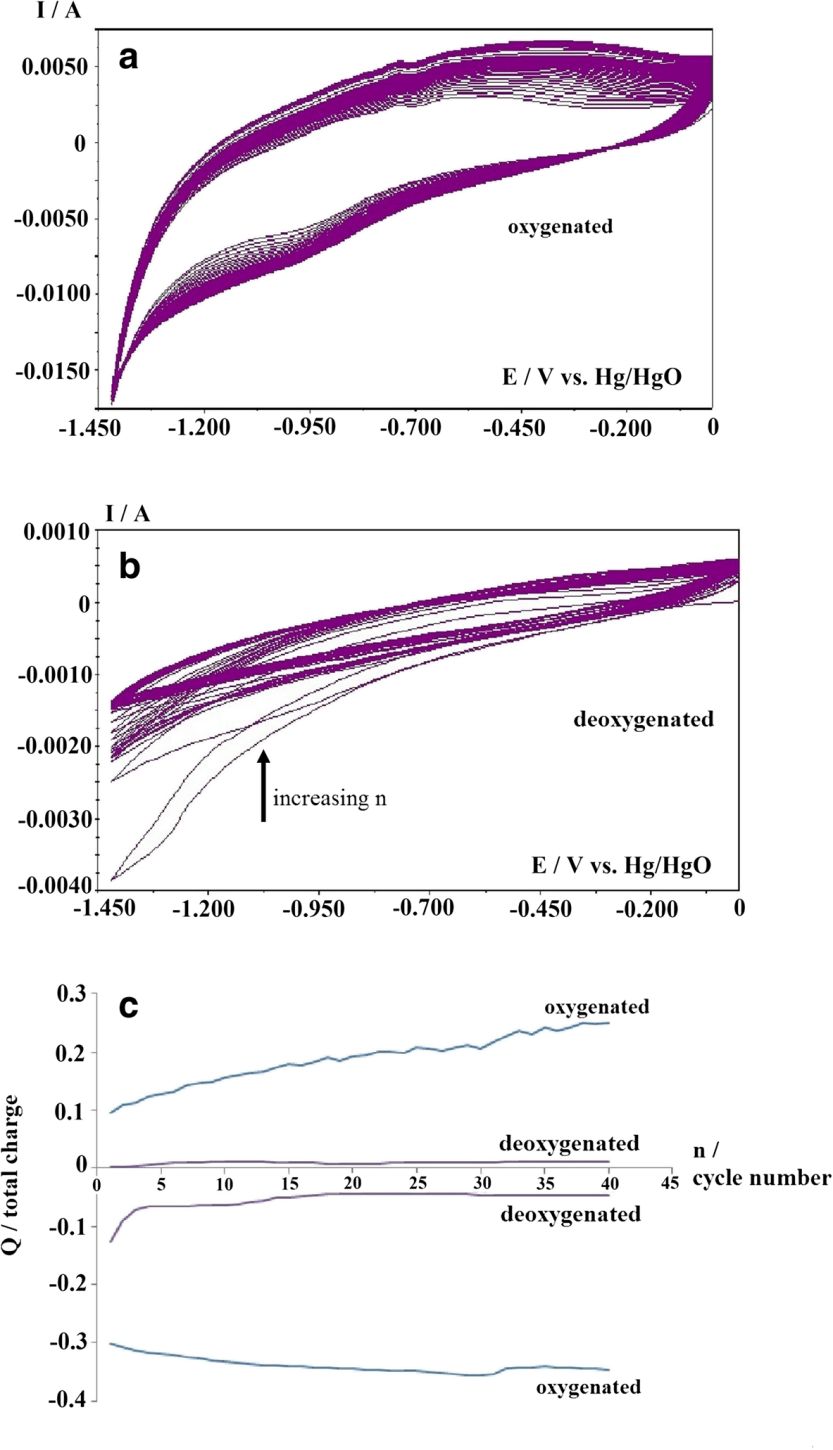
**CV results over 40 cycles at a 25-mV·s**^**-1 **^**scan rate.** For electroreduction of GO to ERGO in 6 M KOH. **(a)** Oxygenated solution, **(b)** deoxygenated solution, and **(c)** total CV charge over 40 cycles for the positive and negative scan in the oxygenated and deoxygenated 6 M KOH solutions.

Figure 1a,b shows the CV plots of GO films over 40 cycles in a 6 M KOH solution at 25 mV·s^-1^ without nitrogen bubbling (oxygenated) and with nitrogen bubbling (deoxygenated), respectively, while Figure 1c shows the charge for the negative and positive scans under both conditions and for each cycle number. It can be observed that the current and charge for both positive and negative scans for the oxygenated solution are higher than those of the deoxygenated solution. This discrepancy is due to the oxygen reduction reaction (ORR) on the GO surface for both the positive and negative scans in the oxygenated condition, which can be expressed as follows:

$$O_{2}+{2H}_{2}O+{4e}^{-}\to 4\mathrm{OH}$$

It should be noted that different types of graphene such as graphene nanosheets [20] and porous graphene [21] are also good electro-catalysts for ORR in lithium-air cells. Graphene-based materials are also finding importance in the ORR such as chemically converted graphene [22], nitrogen-doped graphene [23], polyelectrolyte-functionalized graphene [24], and graphene-based Fe-N-C materials [25]. Therefore, the higher current and charge for each scans for the oxygenated solutions are due to the ORR which occurs concurrent with the reduction of GO to ERGO. When the solution was deoxygenated, the total charge for the negative scan was always higher than the total charge for the positive scan. This trend reveals that there was a net reduction current for each scan that could be attributed to the electrochemical reduction of GO to ERGO in the deoxygenated solution.

### FTIR and Raman spectra

**Figure 2:**
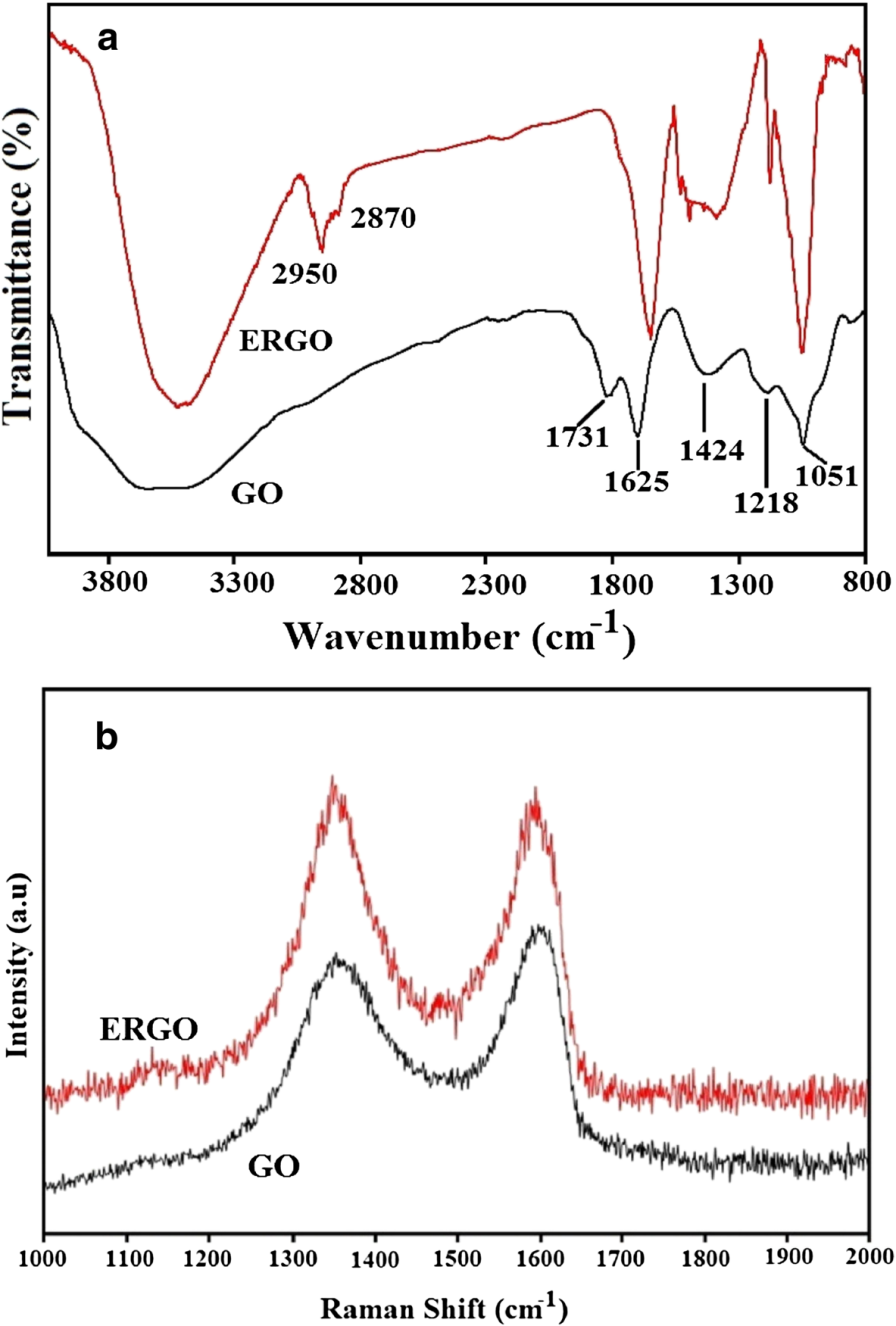
GO and ERGO (a) FTIR spectra and (b) Raman spectra.

Figure 2a shows the FTIR of GO and ERGO films. The FTIR spectrum shows all the characteristic bands for GO: C-O stretching at 1,051 cm^-1^, C-OH stretching at 1,218 cm^-1^, OH bending at 1,424 cm^-1^, stretching of the sp^2^-hybridized C=C bond at 1,625 cm^-1^, C=O stretching at 1,730 cm^-1^, and finally the OH stretching at 3,400 cm^-1^[26]. The FTIR of ERGO retains all characteristic bands of GO, except that the peak of C=O stretching at 1,730 cm^-1^ has completely disappeared, which shows that the C=O functional group in GO was reduced during the voltammetric cycling. The FTIR of ERGO also shows the appearance of new peaks at 2,950 and 2,870 cm^-1^, which are due to the CH_2_ and CH vibrations, respectively. The C=C peak is still present at around 1,610 cm^-1^ which also suggests that the CH_2_ and CH vibrations at 2,950 and 2,870 cm^-1^, respectively, could be due to the reduction of the COOH groups in GO to CH_2_OH.

Figure 2b shows the Raman spectra for GO and ERGO, respectively, where two typical peaks for GO can be found at 1,361 and 1,604 cm^-1^, corresponding to the D and G bands, respectively. The D band is assigned to the breathing mode of A_1g_ symmetry due to the phonon interaction near the K zone boundary, while the G band is attributed to the E_2g_ phonon mode of the sp^2^-bonded carbon atoms [27]. The D and G bands of ERGO were shifted to lower wave numbers of 1,352 and 1583 cm^-1^, respectively, compared to GO. The intensity ratio of the D to G peak (I_D_/I_G_) is an indication of the degree of defects in graphene-related materials where the intensity of the D band is related to the disordered structure of the sp^2^ lattice [13]. For example, pristine graphite which has the lowest disorder density in the sp^2^ lattice gave a ratio of 0.23, while thermally reduced graphene oxide which has the highest disorder density gave a ratio of 1.35 [13]. In this work, the ratio of the I_D_/I_G_ peak for ERGO is 1.03, while the I_D_/I_G_ peak for GO (measured from the nearest baseline) is 1.02. This result is in accordance with previous reports of 1.08 and 1.05 for ERGO and GO, respectively [13]. This result indicates that GO reduction to ERGO did not increase the defect density significantly. It can be suggested that the sp^2^ lattice was maintained even after reduction of GO to ERGO and this is also in accordance with the FTIR of ERGO where the sp^2^-hybridized C=C bonds are still present in ERGO at around 1,610 cm^-1^.

**Figure 3:**
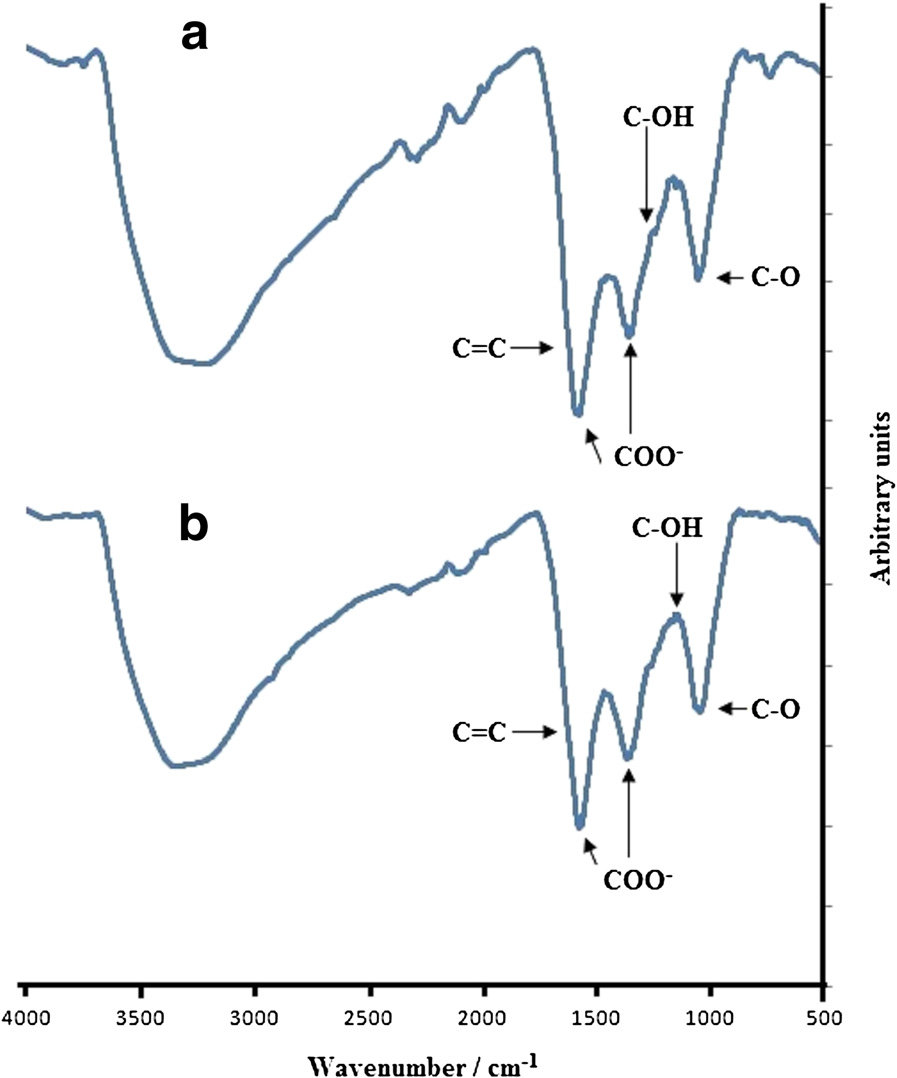
**FTIR of evaporated GO on graphite immersed in deoxygenated 6 M KOH solution. (a)** 1 h **(b)** 4 days.

In order to prove that ERGO is the result of electrochemical reduction of GO in 6 M KOH by voltammetric cycling, GO films were immersed in deoxygenated 6 M KOH solutions for 1 h and 4 days at room temperature. Figure 3a,b shows the FTIR of GO immersed in deoxygenated 6 M KOH for 1 h and 4 days, respectively. The distinct differences shown in these figures and FTIR of pure GO are the disappearance of the C=O peak at 1,730 cm^-1^ and the appearance of two strong new peaks at 1,598 and 1,368 cm^-1^ (for a 1-h immersion) and 1,584 and 1,374 cm^-1^ (for a 4-day immersion). Both peaks (1,598 and 1,584 cm^-1^) and (1,368 and 1,374 cm^-1^) are attributed to the carboxylate COO^-^ group, which has strong vibrations at 1,610 to 1,550 cm^-1^ and 1,420 to 1,300 cm^-1^[28,29]. The presence of the COO^-^ ion is due to the reaction between KOH and the acidic COOH groups in GO. It should be noted that the peaks due to COO^-^ are stronger than the peak due to OH vibration at 3,400 cm^-1^ in the FTIR spectrum of GO immersed in KOH. This is in contrast to the pure GO spectrum where all the peaks are relatively weaker than the OH peak. The complete disappearance of the C=O peak in the FTIR spectrum of GO immersed in KOH also shows that the peak at 1,730 cm^-1^ (C=O) is solely due to the carboxylic COOH group in GO. This also proves that the COOH groups in GO were not reduced to aldehyde HC=O and ketone C=O groups during immersion in 6 M KOH solution. The peaks for the C-OH stretching at 1,218 cm^-1^, OH bending of C-OH at 1,424 cm^-1^, stretching of the sp^2^-hybridized C=C bond at 1,625 cm^-1^ are no longer visible due to the strong vibration of the COO^-^ group in the FTIR spectrum of GO immersed in the KOH solution. In both spectra, vibration at 2,950 and 2,870 cm^-1^ attributed to the CH_2_ and CH, respectively, did not occur, which shows that the C=C was not reduced to CH_2_ and CH, and the COOH was also not reduced to CH_2_OH. Therefore, immersion of GO in deoxygenated 6 M KOH did not reduce GO to RGO, but the ionization of the COOH groups into COO^-^ had taken place at room temperature. However, at higher temperatures (90°C), Fan [30] reported that exfoliated GO can be reduced to graphene in the absence of reducing agents in strong alkaline solutions.

### FESEM and EIS

**Figure 4:**
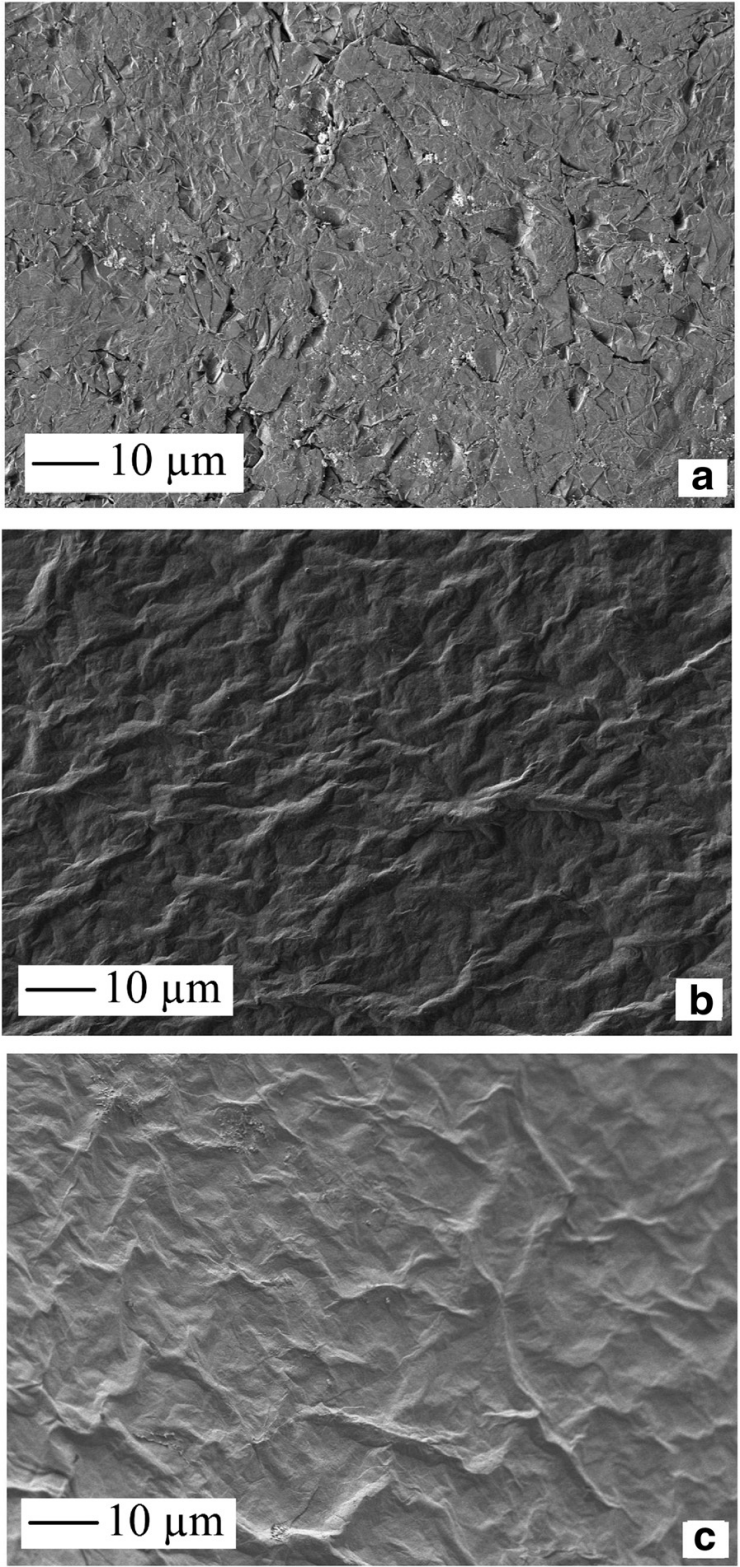
FESEM of (a) graphite surface (b) evaporated GO on graphite, and (c) ERGO on graphite.

Figure 4a,b,c shows the FESEM images of the graphite surface, the evaporated GO films, and ERGO, respectively. It can be seen that the graphite surface consists of compressed flakes of graphite due to the manufacturing process of the material. The FESEM image of the evaporated GO films presents a uniform serrated surface due to the evaporation of the material onto the graphite surface. With GO electroreduction to ERGO in deoxygenated KOH solution, the same surface morphology was maintained as seen in Figure 4c. The GO film was formed from stacked individual layers of GO on the graphite substrate, as the compressed graphite flake surface is no longer visible in Figure 4b,c. Therefore, the electrochemical reduction of the GO film was limited to the surface layer of the film.

**Figure 5:**
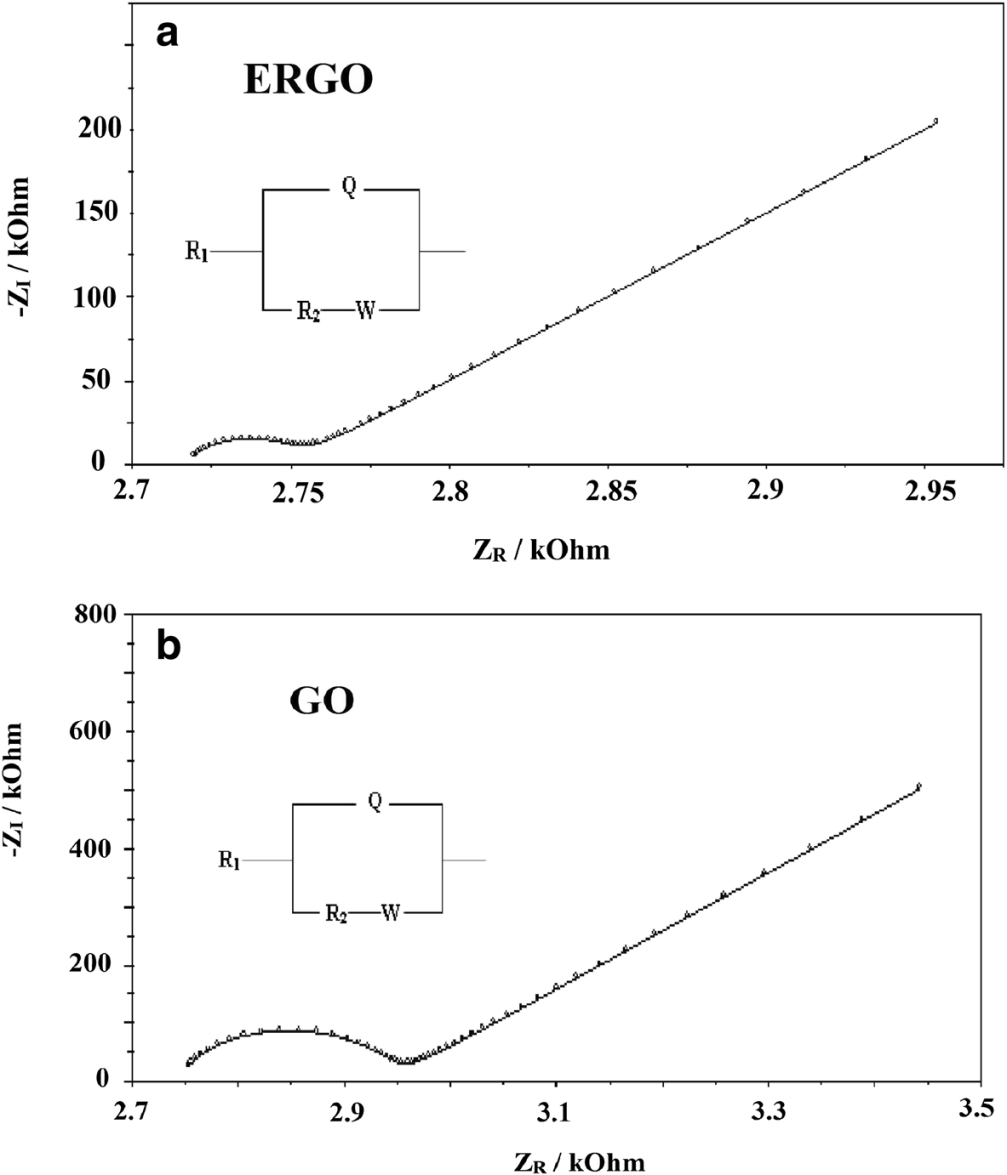
**Nyquist plots in the presence of 23 mM [Fe**^**II**^**(CN)**_**6**_**]**^**3-/4-**^**with 0.1 KCl supporting electrolyte. (a)** GO, and **(b)** ERGO.

Electrochemical impedance spectroscopy were done on both GO and ERGO surfaces in the presence of 23 mM of both [Fe^II^(CN)_6_]^4-^ and [Fe^III^(CN)_6_]^3-^, with 0.1 KCl as the supporting electrolyte. Figure 5a,b shows the Nyquist plots for GO and ERGO, respectively. The Nyquist plots for both GO and ERGO show one semi-circle at higher frequencies which is consistent with the redox reaction of the [Fe^II^(CN)_6_]^4-^ / [Fe^III^(CN)_6_]^3-^ couple across the WE-electrolyte interface. This semi-circle represents the parallel combination of the charge transfer resistance and double-layer capacitance across the electrode-electrolyte interface. The Nyquist plot for GO and ERGO also shows the presence of a Warburg element at lower frequencies which is consistent with the diffusion limiting condition of the redox couple in the solution. The R_1_(Q[R_2_W]) equivalent circuit model was found to accurately fit the experimental data, where an excellent agreement between the experimental data and the simulation of the equivalent circuit model was obtained, with the chi-squared (*x*^2^) value was minimized to 10^-4^. The continuous lines are the simulated data while the symbols represent the experimental data in Figure 5a,b.

The equivalent circuit model can be explained as follows: the *R*_1_ is the solution resistance between the RE-CE and the WE. The *R*_1_ is in series with a parallel arrangement of the constant phase element (CPE - denoted as *Q* in the equivalent circuit) and the *R*_2_-W elements. The CPE was introduced instead of a pure capacitor in the simulations to obtain a good agreement between the experimental and simulation data. The CPE impedance can be defined as *Z*_CPE_ = *Q*^-1^.(*jω*)^-*n*^ where “*n*” is related to the slope of log *Z* vs. log *f* in the Bode plot, *ω* is the angular frequency and *Q* is the combination of properties related to both the surface and the electro-active species, and is independent of frequency. The CPE depends on both the parameter *Q* and the exponent “*n,*” but it should be stressed that *Q* is often approximated to capacitance. The CPE is in parallel arrangement with *R*_2_-W elements, where *R*_2_ is the charge transfer resistance which is in series with the Warburg element W. The circuit diagram is consistent with earlier results using the [Fe^II^(CN)_6_]^4-^ / [Fe^III^(CN)_6_]^3-^ redox couple in solution [13].

For a simple parallel resistance-capacitance combination, the conversion of the CPE parameter into capacitance can be estimated from the following equation [31]:

$$C=Q{\left(\omega_{m,I}\right)}^{n-1}$$

where *C* is the capacitance and *ω*_*m,I*_*= 2πf* and *f* is the frequency at which the imaginary impedance *Z*_*I*_ is maximum, and *Q* is the CPE parameter. Table 1 shows the results from the simulation experiments for both GO and ERGO. It can be seen that ERGO has lower charge transfer resistance compared to GO, which is consistent with previous works [13], where the charge transfer resistance of ERGO and GO is 333 and 831 Ω·cm^2^, respectively. The charge transfer resistance of ERGO reported by Pumera [13] was deposited from GO at a constant potential of -1.2 V vs. Ag/AgCl in phosphate buffer solution at pH 7.2. The I_D_/I_G_ peak for ERGO and GO obtained in this work in the “FTIR and Raman spectra” section is lower than previous report [13], and the FTIR results also shows the presence of the sp^2^ hybridized C=C at around 1,610 cm^-1^ which could explain the lower charge transfer resistance in this work. Clearly, the electrolyte medium and the experimental conditions greatly influenced the charge transfer resistance value of ERGO. This higher charge transfer resistance of ERGO is primarily due to its higher electrical conductivity [32]. The chemical reduction of GO using sodium hydrosulfite to produce RGO also gave an electrical conductivity of seven orders of magnitude higher than GO [33]. The higher electrical conductivity of ERGO could facilitate faster electron transfer to the [Fe^II^(CN)_6_]^4-^/[Fe^III^(CN)_6_]^3-^ redox couple, thus ERGO has lower charge transfer resistance R_2_ compared to GO. The higher charge transfer resistance of GO compared to ERGO in Table 1 has a good correlation with the higher electrical resistivity of GO compared to RGO obtained by Zhou [33]. It can be seen also that the value of surface capacitance for ERGO is nearly five times higher compared to that for GO. This could be due to the electrochemical reduction of the C=O and epoxy C-O-C in the GO to produce more polarized C-OH groups in ERGO, thus increasing the ion accumulation on the ERGO surface. The lower capacitance of GO compared to ERGO is also in accordance with previous reports, thus GO is not useful for supercapacitor applications [34-38].

**Table 1:** Parameters of GO and ERGO obtained using EIS

**WE**	***Q *** **(S·s**^***n***^**)**	***n***	***R***_**2 **_**(Ω·cm**^**2**^**)**	***W *** **(S·s**^**1/2**^**)**	***C *** **(F cm**^**-2**^**)**
GO	1.5 × 10^**-**6^	0.9096	196.9	1.99 × 10^**-**3^	6.66 × 10^**-**7^
ERGO	8.04 × 10^**-**6^	0.9100	32.7	3.47 × 10^**-**3^	3.30 × 10^**-**6^

### Cyclic voltammetry in [Fe^II^(CN)_6_]^3-/4-^ redox couple

**Figure 6:**
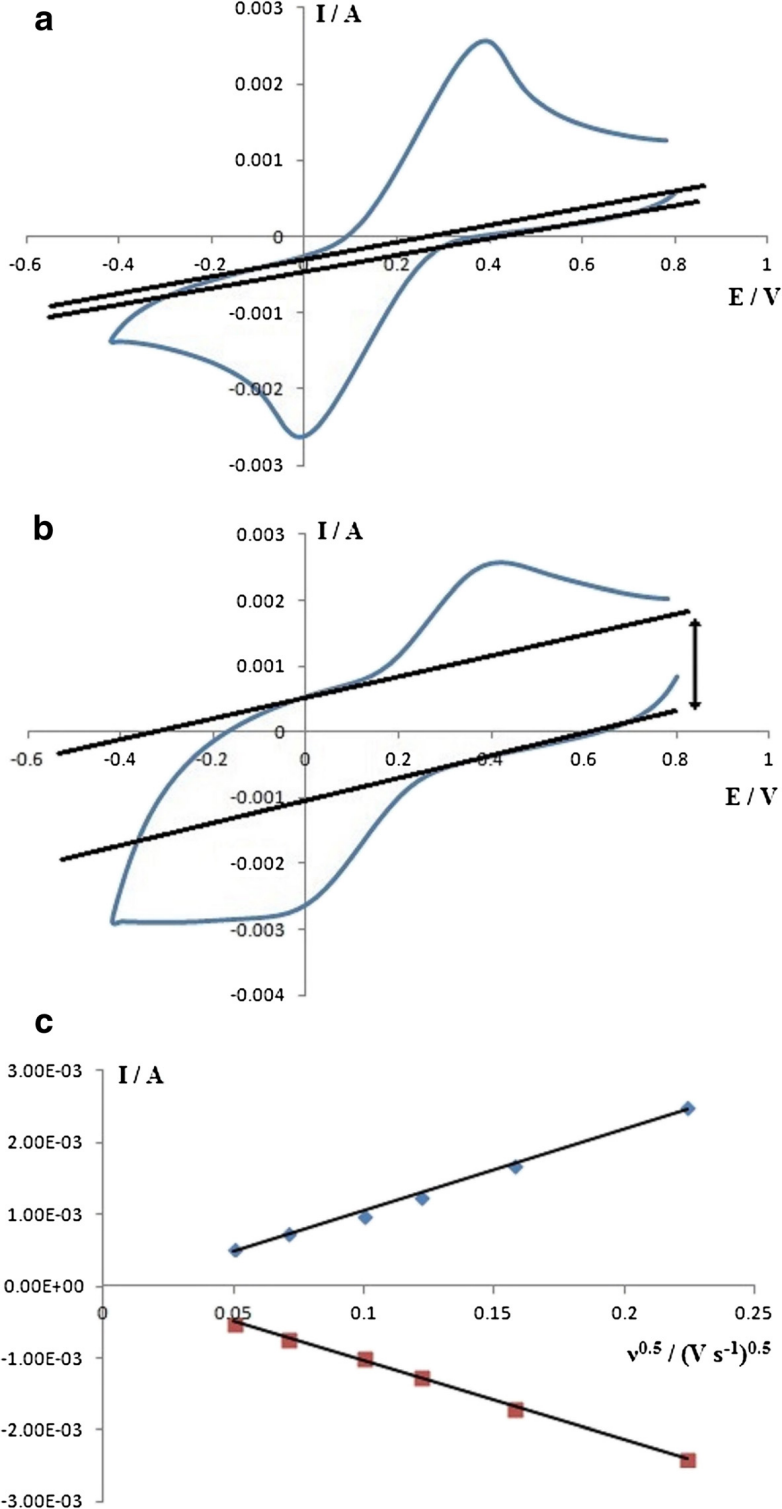
**Cyclic voltammetry at 50 mV·s**^**-1 **^**with 23 mM [Fe**^**II**^**(CN)**_**6**_**]**^**4 **-^**/ [Fe**^**III**^**(CN)**_**6**_**]**^**3 **-^**redox couple and *I *vs. *v***^**1/2 **^**plots.** Cyclic voltammetry in 0.1-M KCl supporting electrolyte **(a)** GO and **(b)** ERGO and **(c)** I vs. ν^1/2^ plots of GO.

Cyclic voltammetry with the [Fe^II^(CN)_6_]^4-^/[Fe^III^(CN)_6_]^3-^ redox couple in 0.1 M KCl supporting electrolyte was done on both the GO and ERGO films with a SCE as the reference. Figure 6a,b shows the voltammetric reponse for GO and ERGO films at 50 mV·s^-1^. In Figure 6a, the anodic and cathodic currents of the redox couple for the GO film has almost similar baseline currents, as shown by the two straight lines very close to each other. The baseline for the anodic and cathodic currents has larger separation for ERGO films as shown in Figure 6b. This is due to the larger surface capacitance of the highly polarized ERGO surface, which was mentioned earlier in the “FESEM and EIS” section. Both anodic and cathodic currents for the GO film show straight lines from the plots of *I* vs. *ν*^1/2^ as shown in Figure 6c. From the Randles-Sevcik equation $=0.4463\mathrm{nF}{\left(\frac{\mathrm{nF}}{\mathrm{RT}}\right)}^{1/2}\mathrm{Ac}D^{1/2}v^{1/2}$, the diffusion coefficient (*D*) of the [Fe^II^(CN)_6_]^4-^/[Fe^III^(CN)_6_]^3-^ redox couple in 0.1 M KCl was estimated to be 5.9 × 10^-10^ m^2^·s^-1^.

## Conclusion

Solid-phase electrochemical reduction of GO films on graphite in alkaline solution produced ERGO which was confirmed with FTIR and Raman spectra. The EIS results obtained using [Fe^II^(CN)_6_]^4-^/[Fe^III^(CN)_6_]^3-^ redox couple in 0.1-M KCl supporting electrolyte indicated that the charge transfer resistance for ERGO is lower than GO and is consistent with the higher electrical conductivity of ERGO. The results also reveal that the capacitance of ERGO is larger than GO, due to its higher polarity of ERGO. This result is also supported by voltammetry of both GO and ERGO in [Fe^II^(CN)_6_]^4-^/ [Fe^III^(CN)_6_]^3-^ redox couple in 0.1-M KCl supporting electrolyte, where ERGO surface has a larger separation of the anodic and cathodic baseline currents due to the larger capacitance compared to the GO surface.

## Competing interests

The authors declare that they have no competing interests.

## Authors’ contributions

MS and SB synthesized and characterized GO. ME and MRM ran experiments of CV and EIS. WJB wrote the manuscript. All authors read and approved the final manuscript.
